# Low Serum LH Levels During Ovarian Stimulation With GnRH Antagonist Protocol Decrease the Live Birth Rate After Fresh Embryo Transfers but Have No Impact in Freeze-All Cycles

**DOI:** 10.3389/fendo.2021.640047

**Published:** 2021-04-23

**Authors:** Yiyang Luo, Shan Liu, Hui Su, Lin Hua, Haiying Ren, Minghui Liu, Yuting Wan, Huanhuan Li, Yuan Li

**Affiliations:** ^1^ Medical Center for Human Reproduction, Beijing Chao-Yang Hospital, Capital Medical University, Beijing, China; ^2^ Department of Hysteroscopic Center, Beijing Fu-Xing Hospital, Capital Medical University, Beijing, China; ^3^ Department of Biomedical Information, School of Biomedical Engineering, Capital Medical University, Beijing, China

**Keywords:** luteinizing hormone, cumulative live birth rate, controlled ovarian stimulation, GnRH antagonist, fresh embryo transfer, frozen-thawed embryo transfer

## Abstract

**Objective:**

To explore the association between serum LH levels and the cumulative live birth rate (CLBR) within one complete cycle, and the impact of serum LH levels on the live birth rate (LBR) after the initial embryo transfer (ET) considering different ET strategies (fresh or freeze-all).

**Design:**

A retrospective cohort study.

**Setting:**

University-affiliated reproductive center.

**Patients:**

1480 normogonadotrophic women who underwent COS with GnRH antagonist protocol for the first IVF/ICSI attempt.

**Intervention(s):**

The sample was stratified into low and higher LH groups according to serum LH peak levels of <4 (Group A) and ≥4 IU/L (Group B) during COS. Patients were also sub-grouped into conventional fresh/frozen ET cycles and freeze-all cycles.

**Main outcome measure(s):**

The LBR after the initial embryo transfer and the CLBR within one complete cycle.

**Secondary outcome measure(s):**

The numbers of day-3 high-quality embryos, the numbers of embryos available, and the other pregnancy outcomes after the initial ET.

**Result(s):**

In the whole cohort, the CLBRs decreased significantly in the low (63.1% vs. 68.3%, *P*=.034) LH group compared to the higher LH group. Subgroup analysis revealed that patients with low LH levels had lower LBR after fresh ET (38.0% vs. 51.5%, *P*=.005) but comparable LBR after the first frozen-thawed ET (FET) in freeze-all cycles (49.8% vs. 51.8%, *P*=.517) than patients with higher LH peak levels. Likewise, patients with low LH levels had lower CLBR for conventional fresh/frozen ET cycles (54.8% vs. 66.1%, *P*=.015) but comparable CLBR for the freeze-all cycles (66.8% vs. 69.2%, *P*=.414) than those with higher LH levels. Following confounder adjustment, multivariable regression analyses showed that low LH level was an independent risk factor for the CLBR in the whole cohort (odds ratio (OR): 0.756, 95% confidence interval (CI): 0.604-0.965, *P*=.014) and in patients who underwent the conventional ET strategy (OR: 0.596, 95% CI: 0.408-0.917, *P*=.017). Moreover, the adverse impact of low LH levels on LBRs maintained statistically significant after fresh transfers (OR: 0.532, 95% CI: 0.353-0.800, *P*=.002) but not after the first FETs in freeze-all cycles (OR: 0.918, 95% CI: 0.711-1.183,* P*=.508).

**Conclusion(s):**

In comparison with higher LH levels, low LH levels decrease the CLBRs per oocyte retrieval cycle for normogonadotrophic women who underwent COS using GnRH antagonists. This discrepancy may arise due to the significant detrimental effect of low LH levels on the LBRs after fresh embryo transfers.

## Introduction

Currently, the gonadotropin-releasing hormone (GnRH) antagonist protocol has already been one of the mainstream controlled ovarian stimulation (COS) protocols because of its convenience, safety, and comparable efficacy compared with the classical GnRH agonist long protocol (1). GnRH antagonists act by rapidly and reversibly competing for pituitary GnRH receptors. Various endogenous LH levels can be induced by different administration times or doses of GnRH antagonists in IVF/ICSI cycles (2–4). Moreover, patients may respond diversely to the same antagonist regimen. Consequently, LH levels and variations in GnRH antagonist stimulation cycles present individual differences (5, 6).

Luteinizing hormone (LH) not only plays a central role in follicle development, ovulation, and steroidogenesis (7) but also influences embryo implantation and corpus luteum function (8, 9). Shoham (10) proposed a clinical therapeutic window for LH in COS. However, the optimal range of LH in COS is not yet well understood, and debates focused on the predictive value of LH levels for treatment outcomes never cease. Heterogeneity is prevalent in the existing studies, such as in the stimulation protocol, patient characteristics, day of LH measurement, and cut-off values for LH (11–17).

Concerning the LH threshold during COS using GnRH antagonists, by setting an absolute LH value or LH quartiles on a fixed predefined day, some scholars conclude that LH concentrations do not influence cycle outcomes (12, 15, 16). Inversely, clinical evidence from multiple LH measurements revealed that low LH levels were associated with increased early pregnancy loss (11, 17, 18). While the supplementation of LH activity may help improve the pregnancy outcomes for patients of LH overinhibited (19, 20).

To date, the number of studies investigating the effect of serum LH concentrations on pregnancy outcomes in GnRH antagonist-treated cycles remains limited. There is not yet a report about the association between LH levels and cumulative live birth rates (CLBRs), and little is known about the influence of LH levels on live birth rates (LBRs) when performing different embryo transfer (ET) strategies. This study’s objective was to address these considerations.

## Materials and Methods

In this retrospective cohort study, we obtained data from patients who underwent COS using GnRH antagonist protocol for the first *in vitro* fertilization/intracytoplasmic sperm injection (IVF/ICSI) attempt from January 2017 to June 2019 at the Medical Center for Human Reproduction, Beijing Chao-Yang Hospital affiliated with Capital Medical University. The Ethics Committee of Chao-Yang Hospital approved this study. All women provided written informed consent.

All the patients enrolled were with good ovarian reserve, stimulated with GnRH antagonist protocol followed by either fresh ET or cryopreservation of all embryos, and were followed up until the treatment cycle was completed. Namely, all the embryos were used up, or a live birth was achieved. The inclusion criteria were as follows: (1) patients with good ovarian reserve, meaning age ≤38 years, basal serum FSH <10 IU/L, and antral follicle count (AFC) ≥6, (2) regular menstrual cycle, 21-35d, (3) body mass index (BMI) <30 kg/m^2^, (4) at least one embryo was available. The exclusion criteria were as follows: patients with a diagnosis of polycystic ovary syndrome, diabetes mellitus, hypogonadotropic amenorrhea, genital system tumors, abnormal uterine cavity morphology (i.e., Müllerian malformations, submucosal myoma, severe intrauterine adhesion, adenomyosis), or those who underwent preimplantation genetic testing. We also excluded the patients who happened a premature LH surge.

### Ovarian Stimulation

Ovarian stimulation was initiated with an individualized dose of 150-225 IU recombinant FSH (rFSH: Gonal-F^®^, Merck Serono) on day 3 of the menstrual cycle. Gonadotrophin dosage adjustment was allowed according to the follicular development monitored by serial transvaginal ultrasound scans and hormone measurements after 4-5 days of fixed-dose rFSH. Pituitary downregulation was performed with a flexible GnRH antagonist protocol. In brief, 0.125-0.25 mg cetrorelix acetate (Cetrotide^®^, Merck Serono, Geneva, Switzerland) was given when the leading follicular diameter ≥14 mm since stimulation day 5. The dosage and duration adjustment of the antagonist was allowed in terms of the clinician’s experience and discretion, based on the patient’s characteristics, follicular development, and subsequent LH levels. Recombinant LH (rLH: Leuveris^®^, Merck Serono) was supplemented when the follicles’ growth is slow or not synchronized with the hormone measurements from the day of GnRH-antagonist administered. Triggering of final oocyte maturation was performed with 0.2 mg of triptorelin (Decapeptyl^®^, Ferring) plus 2000 IU recombinant hCG (Ovitrelle^®^, Merck Serono) as soon as at least three follicles of 17 mm were visible, followed by ovum pick-up 34-36 hours later. The retrieved oocytes were fertilized by IVF or ICSI according to the status of the sperm.

### Blood Samples and Hormone Assays

As a routine clinical procedure in our center, all blood samples were drawn early in the morning, between 8 am and 10 am. Serum hormone profiles were measured as follows: (i) on the initial day of stimulation cycle, (ii) 4-5 days after the gonadotrophins administration, (iii) then every 1 to 2 days according to the individual follicular development and endocrine profile until the day of triggering. We recorded the LH peak levels and times of LH lower than 1.2 IU/L during the entire stimulation.

The hormone levels were analyzed at the central laboratory of Chao-Yang Hospital with an electrochemiluminescence immunoassay kit (Roche Diagnostics GmbH, Mannheim, Germany). The detection limits were 0.1 IU/L for FSH and LH, 5.00 pg/mL for E_2_, and 0.05 ng/mL for P. The inter-assay and intra-assay coefficients of variation were 2.46~4.55% and 5.10%~8.11% for E_2_, 3.78%~5.66% and 3.78%~5.92% for P, 3.79%~5.48% and 2.26%~5.16% for FSH, and 3.16%~5.66% and 3.12%~4.67% for LH, respectively.

### Embryo Transfer and Luteal Phase Support

Two good-quality cleavage embryos were routinely transferred or vitrified on the third day after ovum pick-up, and the remaining embryos were cultured for 2-3 more days for blastocyst vitrification. A good-quality embryo was defined as follows: the number of cells on day 3 was 7-9 cells, <20% fragmentation, and regular-sized cells. Freeze-all procedures were performed only in patients with a high risk of ovarian stimulation syndrome, serum P level exceeding 1.5 ng/mL during COS, or those with inadequate endometrial morphology or thickness. If available, up to two cleavage embryos were transferred in the first frozen-thawed embryo transfer (FET) cycle. Luteal phase support was administered in both fresh transfer and freeze-all cycles until 9-10 weeks after conception as described previously (21).

### Outcome Assessment

This study’s primary outcomes were the LBR after the initial ET (i.e., fresh ET or the first FET in the freeze-all cycle) and the CLBR within one complete treatment cycle. We defined the LBR as the delivery of a live infant born after 24 completed weeks of gestation. The secondary outcomes included the numbers of day-3 high-quality embryos, the numbers of embryos available, and the other pregnancy outcomes after the initial ET. We defined biochemical pregnancy as serum β-hCG level >15 IU/L at 12-14 days after embryo transfer. The implantation rate was calculated as the number of visible gestational sacs divided by the number of embryos transferred. We defined the clinical pregnancy as a pregnancy diagnosed by ultrasonographic visualization of one or more gestational sacs or definitive clinical signs of pregnancy at 7-8 gestational weeks and the early pregnancy loss as spontaneous pregnancy loss before 12 gestational weeks. These definitions are in accordance with the latest revision of “The International Glossary on Infertility and Fertility Care, 2017” (22).

### Statistical Methods

Patients were stratified into two groups according to serum LH peak levels below or above 4 IU/L during the entire COS period. Comparisons between groups were carried out using the Student’s *t* test, Mann-Whitney test, Pearson chi-square test, or Fisher’s exact test, as appropriate. We performed multivariate logistic regression analyses to identify the LH effect on the cumulative live births (CLBs) within one complete cycle and the live births (LBs) after the initial ET. Univariate regression analyses were conducted to identify candidate factors correlated with CLBs and LBs. The candidate variables were as follows: LH category (the higher LH group was taken as reference), ET strategy (conventional fresh/frozen ET vs. freeze-all policy), the total dose of GnRH antagonists and rLH supplementation, female age and BMI at COS, duration and type of infertility, and the number of oocytes obtained. The hormone profiles on stimulation day one and trigger day, total dose and days of gonadotropins, the number of embryos transferred, embryo stage at transfer (cleavage vs. blastocyst), and the endometrial thickness were also included. We only included variables that show a tendency of association with CLBs or LBs in the univariate analysis (*P*<.25) in the final multivariate model. All independent variables were entered into the final multivariant logistic regression model with the forward (LR) method. The likelihood of CLB and LB was presented as the odds ratio (OR) and 95% confidence interval (CI). All statistical analyses were performed with the Statistical Package for Social Sciences (SPSS, ver. 25.0). Two-sided tests with a *P*-value of <.05 were considered to be statistically significant.

## Results

**Figure 1:**
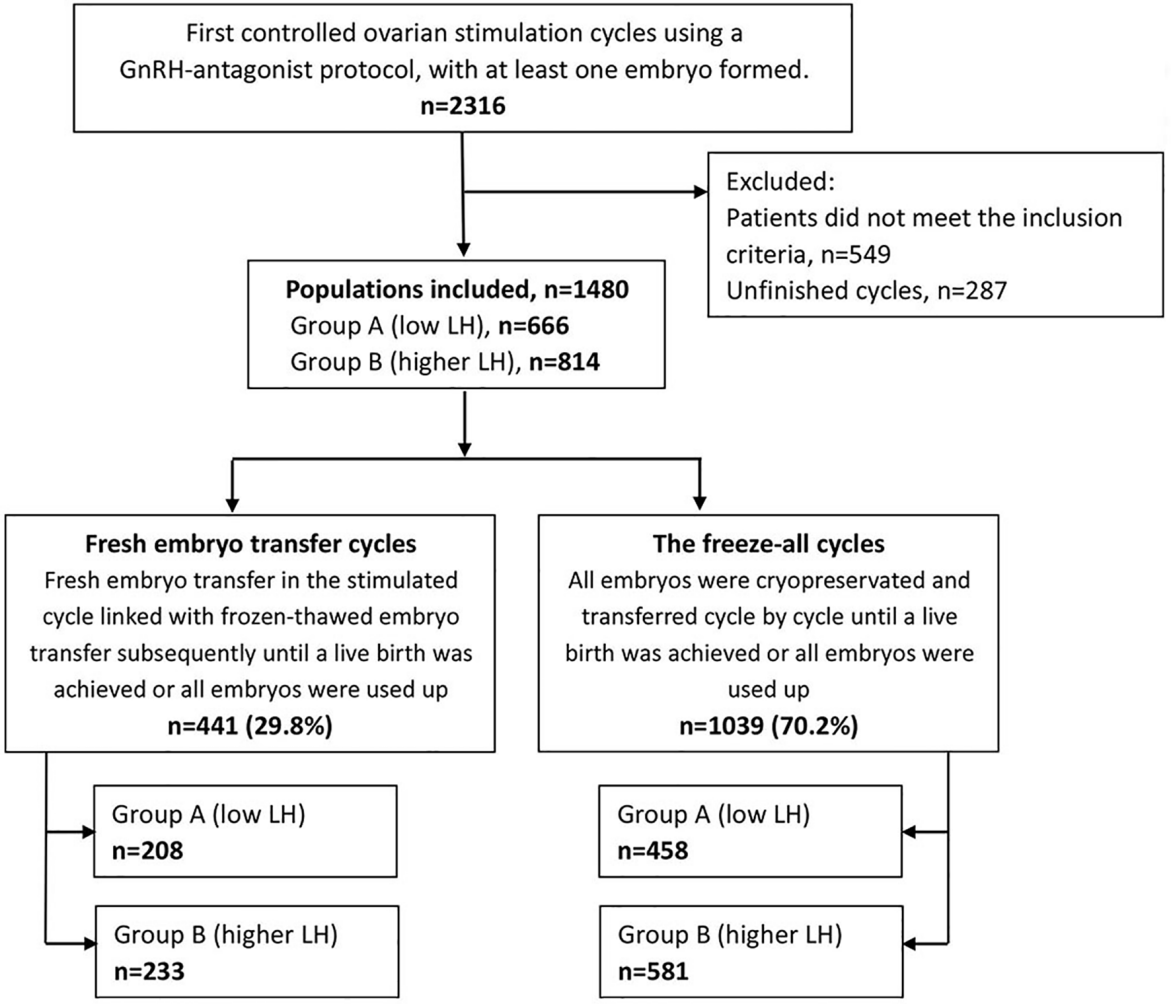
Flow chart and group division of this study.

The methods of searching and group divisions are shown in **Figure 1**. Overall, 1480 patients were included in this research. Among them, 441 patients underwent fresh ETs, and 1039 patients received the freeze-all policy. Patients of fresh ET cycles or the freeze-all cycles distributed similarly in the low and higher LH groups (*P*=.275).

### Patients and Cycle Characteristics

The patients’ baseline characteristics are summarized and described in **Table 1**. Patients in the low LH group were slightly younger than those in the higher LH group, with statistical significance. Comparisons between groups did not reveal any significant differences in BMI, AFC, baseline FSH levels, type and duration of infertility, or IVF treatment indications.

**Table 1:** Baseline patient characteristics.

Characteristics	Group A	Group B	*P*
	n=666	n=814	
Age (y), mean ± SD	31.48 ± 3.61	32.18 ± 3.77	<0.001
BMI (kg/m^2^), mean ± SD	22.81 ± 3.41	22.84 ± 3.62	0.857
AFC, mean ± SD	15.80 ± 7.61	15.97 ± 7.63	0.675
Hormone profile at baseline, mean ± SD			
E_2_ (pg/mL)	47.95 ± 19.00	49.20 ± 18.34	0.201
FSH (IU/L)	6.50 ± 2.12	6.77 ± 1.90	0.163
LH (IU/L)	3.90 ± 2.67	4.75 ± 2.29	<0.001
Infertility diagnosis, n (%)			0.303
Primary	403 (60.5%)	471 (57.9%)	
Secondary	263 (39.5%)	343 (42.1%)	
Duration of infertility (y), median (IQR)	2.5 (2-4)	3 (2-4)	0.942
Cause of infertility, n (%)			0.962
Male factor	56 (8.4%)	69 (8.5%)	
Female factor	314 (47.1%)	375 (46.1%)	
Combined factors	241 (36.2%)	305 (37.5%)	
Unexplained factor	55 (8.3%)	65 (8.0%)	

BMI, body mass index; AFC, antral follicle count; E_2_, estradiol; FSH, follicle-stimulation hormone; LH, luteinizing hormone.

COS and IVF/ICSI-ET parameters per LH group in the whole population are provided in **Table 2**. The total dose of antagonists was significantly less in the low LH group than in the higher LH group. The rLH supplementation dose was more in the low LH group than in the higher LH group with statistical significance. However, the days and total dose of gonadotrophins, insemination method, fertilization rate, number of oocytes retrieved, number of MII oocytes, number of day-3 good-quality embryos, and number of embryos available were similar between groups. Endometrial thickness at transfer, the number of embryos transferred, and the type of embryo transferred in the initial ET cycles were also comparable between groups. Comparisons between different LH groups in fresh transfer cycles or freeze-all cycles demonstrated the same trend as in overall cycles (**Supplemental Table 1**).

**Table 2:** Parameters of ovarian stimulation and embryo transfer.

Characteristics	Group A	Group B	*P*
	n=666	n=814	
Duration of stimulation (days)	9.86 ± 1.46	9.77 ± 1.40	0.223
Total gonadotrophin dose (IU)	2297.54 ± 788.52	2327.99 ± 804.57	0.465
Total antagonist dose (mg)	0.375 (0.125-0.5)	0.5 (0.25-0.625)	<0.001
rLH supplementation dose (IU)	375 (150-900)	300 (75-750)	<0.001
Percentage of rLH supplementation	561 (84.2%)	614 (75.4%)	<0.001
Hormone profile at trigger day			
E_2_ (pg/mL)	3614.13 ± 2118.49	3583.40 ± 2257.76	0.789
P (ng/mL)	0.91 ± 0.58	0.96 ± 0.69	0.229
LH (IU/L)	2.11 ± 1.01	3.13 ± 1.92	<0.001
Frequencies of LH below 1.2 IU/L			
0	268 (40.2%)	495 (60.8%)	<0.001
1	224 (33.6%)	246 (30.2%)	0.161
≥2	174 (26.1%)	73 (9.0%)	<0.001
FORT^a^	0.54 ± 0.25	0.52 ± 0.24	0.091
IVF	454 (68.2%)	537 (66.0%)	0.371
ICSI or IVF - ICSI split	212 (31.8%)	277 (34.0%)	
Fertilization rate (IVF)	0.62 ± 0.21	0.60 ± 0.24	0.069
Fertilization rate (ICSI)	0.74 ± 0.20	0.76 ± 0.21	0.256
No. of oocytes retrieved	14.46 ± 6.96	14.21 ± 7.17	0.492
No. of MII oocytes	9.84 ± 5.62	9.56 ± 5.56	0.589
No. of good-quality embryos on day 3	4.29 ± 3.49	4.23 ± 3.50	0.769
No. of total embryos available	4.23 ± 2.41	4.18 ± 2.38	0.691
**Parameters of the first ET cycle**			
Fresh ET	208 (31.2%)	233 (28.6%)	0.275
Frozen-thawed ET	458 (68.8%)	581 (71.4%)	
Endometrial thickness	9.56 ± 1.96	9.42 ± 1.87	0.162
No. of embryos transferred	1.95 ± 0.31	1.97 ± 0.32	0.316
Cleavage embryos transferred	599 (89.9%)	731 (89.8%)	0.931
Blastocyst	67 (10.1%)	83 (10.2%)	

Data are mean ± SD, median (IQR), or n (%). E_2_, estradiol; P, progesterone; rLH, recombinant LH; MII, metaphase II; ET, embryo transfer.

a FORT= follicle output rate, calculated as the number of 16-22 mm preovulatory follicles/the number of 3-8 mm antral follicles on the third day of the menstrual cycle.

We also divide patients according to the ovarian response. **Supplemental Figure 1** depicts the distribution of the categories of the number of oocytes retrieved (<4, 4-9, 10-15, >15). No significant differences in the distribution were observed between the low and higher LH groups either after fresh ETs (*P*=.982) or freeze-all cycles (*P*=.108). The proportion of oocytes retrieved were most in 4-9 and 10-15 subgroups in fresh ET cycles (47.6% and 36.2%, respectively), but were most in >15 and 10-15 subgroups in freeze-all cycles (49.4% and 30.5%, respectively).

### Pregnancy Outcomes in the IVF/ICSI Cycles

**Figure 2:**
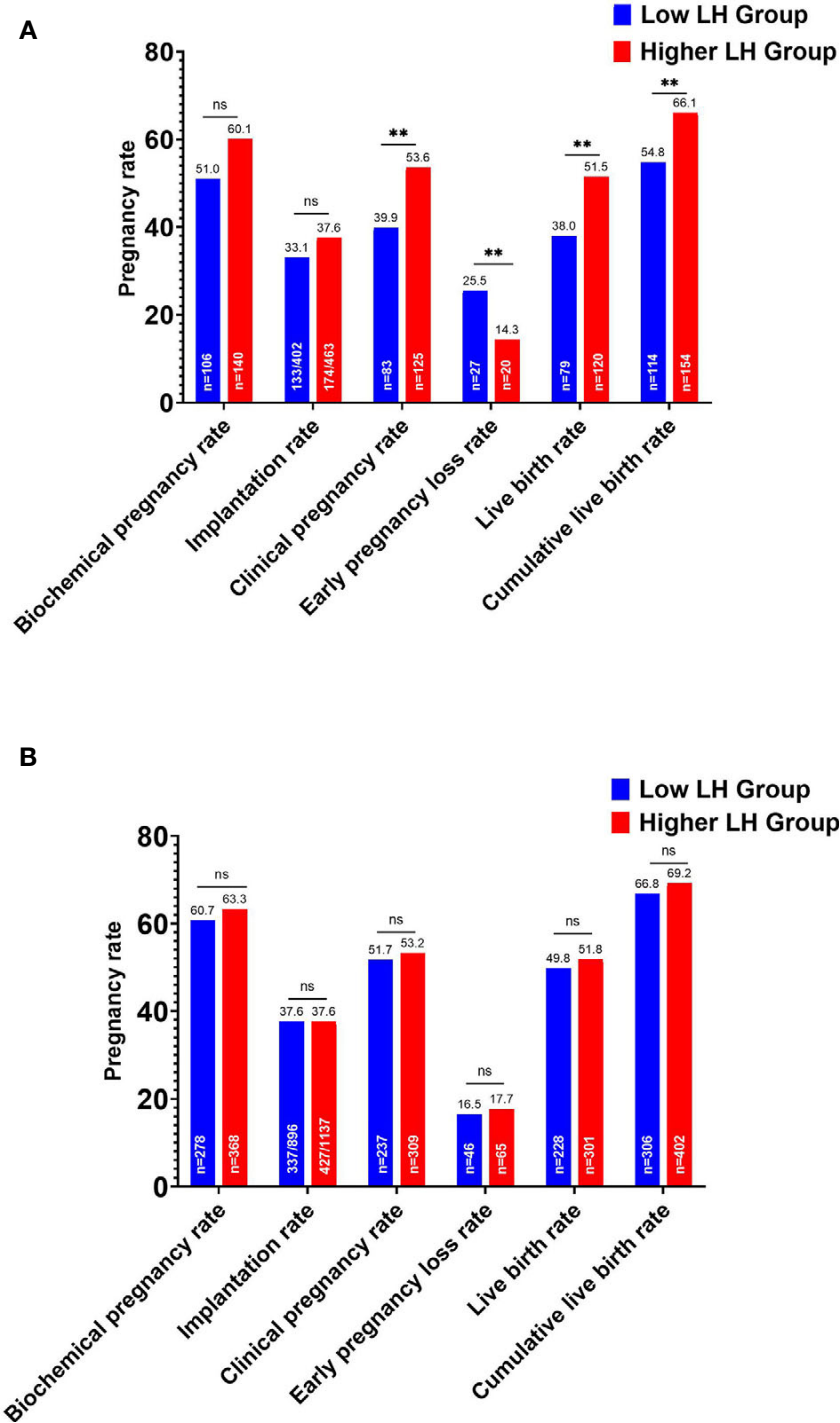
Pregnancy outcomes of the low and higher LH groups stratified by embryo transfer strategy. **(A)** Pregnancy outcomes after fresh embryo transfers and the cumulative live birth rate for the conventional embryo transfer policy. **(B)** Pregnancy outcomes after the first frozen-thawed embryo transfers in freeze-all cycles and the cumulative live birth rate for freeze-all policy. ^**^ *P* < .05. ns, no statistical significance.

In the whole cohort, the overall CLBR per oocyte retrieval cycle decreased significantly in the low LH group than in the higher LH group (420/666, 63.1% vs. 556/814, 68.3%, *P*=.034). According to the ET strategy, further division of the study population showed that the LBR and clinical pregnancy rate of the low LH group were significantly lower in fresh ET cycles, with an elevated early pregnancy loss rate (**Figure 2**). The CLBR of the conventional fresh/frozen ET cycles was significantly lower in the low LH group than in the higher LH group (**Figure 2**). However, no significant differences in these pregnancy outcomes were identified in freeze-all cycles (**Figure 2**).

The pregnancy outcomes of patients with a different ovarian response in the low and higher LH groups after different ET strategies were summarized and described respectively in **Table 3**. Patients in the higher LH group had improved LBRs in patients with suboptimal (the number of oocytes retrieved 4-9), optimal (the number of oocytes retrieved 10-15), and high ovarian response (the number of oocytes retrieved >15) than those in the low LH group after fresh ETs. In suboptimal ovarian responders, the difference was statistically significant (36.7% vs. 50.9%, *P*=.039). Likewise, the CLBRs for the conventional fresh/frozen ET cycles by the number of oocytes retrieved were more in the higher LH group in all subgroups, but with no significant differences. However, no such disparity was seen after the initial FETs in freeze-all cycles.

**Table 3:** Pregnancy outcomes of the low and higher LH groups based on different ovarian responses and embryo transfer strategies.

Oocytes Count	4-9				10-15				>15			
	Fresh cycle, N=210		Freeze-all cycle, N=209		Fresh cycle, N=159		Freeze-all cycle, N=317		Fresh cycle, N=59		Freeze-all cycle, N=513	
	Group A, n=98	Group B, n=112	Group A, n=86	Group B, n=123	Group A, n=77	Group B, n=82	Group A, n=129	Group B, n=188	Group A, n=27	Group B, n=32	Group A, n=243	Group B, n=270
Biochemical pregnancy rate, % (n)	51.0% (50)	59.8% (67)	50.0% (43)	54.5% (67)	51.9% (40)	59.8% (49)	52.7% (68)	64.9% (122)^c^	55.5% (15)	62.5% (20)	68.7% (167)	66.3% (179)
Implantation rate, % (n/N)	35.3% (65/184)	37.5% (84/224)	26.8% (44/164)	31.7% (73/230)	32.1% (50/156)	37.2% (61/164)	34.3% (87/254)	38.0% (132/347)	30.2% (16/53)	38.1% (24/63)	43.1% (206/478)	41.6% (222/534)
Clinical pregnancy rate, % (n)	40.8% (40)	53.5% (60)^b^	38.4% (33)	48.0% (59)	39.0% (30)	52.4% (43)^b^	47.3% (61)	53.2% (100)	44.4% (12)	56.3% (18)	58.8% (143)	55.6% (150)
Early pregnancy loss rate, % (n)	28.0% (14)	14.9% (10)^b^	27.9% (12)	14.9% (10)^b^	25.0% (10)	16.3% (8)	11.8% (8)	21.3% (26)	20.0% (3)	10% (2)	18.0% (30)	16.2% (29)
Live birth rate, % (n)	36.7% (36)	50.9% (57)^a^	36.0% (31)	46.3% (57)	38.9% (30)	50.0% (41)	46.5% (60)	51.1% (96)	44.4% (12)	56.3% (18)	56.4% (137)	54.8% (148)
Cumulative live birth rate, % (n)	46.9% (46)	59.8% (67)^b^	45.3% (39)	54.5% (67)	61.0% (47)	68.2% (56)	60.5% (78)	63.3% (119)	74.1% (20)	84.4% (27)	77.8% (189)	80.0% (216)

^a^P=0.039; ^b^P<0.1; ^c^P=0.030.

The results of multivariate logistic regression analyses are presented in **Table 4**. In the whole cohort, after adjustment for all the potential confounders, the low LH levels showed a detrimental influence on the CLBs with statistical significance (odds ratio (OR): 0.756, 95% confidence interval (CI): 0.604-0.965, *P*=.014). Subgroup analyses demonstrated that after confounder-adjustment with multivariable regression analysis, the low LH effect was a risk factor for LBs after fresh ETs (OR: 0.532, 95% CI: 0.353-0.800, *P*=.002), but this adverse effect no longer remained statistically significant after the first FETs in freeze-all cycles (OR: 0.918, 95% CI: 0.711-1.183, *P*=.508). Likewise, the low LH level was an independent risk factor for the CLBR in patients who underwent the conventional ET strategy (OR: 0.596, 95% CI: 0.408-0.917, *P*=.017). However, this adverse effect no longer exists on CLBR for the freeze-all cycles.

**Table 4:** Multivariate logistic regression analysis by treatment outcome.

Outcome	Variable	COR (95% CI)	P value	AOR (95% CI)	P value
**CLBR^a^**	Low LH^*^	0.792 (0.638, 0.983)	0.034	0.756 (0.604, 0.965)	0.014
	No of oocytes retrieved	1.090 (1.070, 1.110)	<0.001	1.091 (1.071, 1.111)	<0.001
**LBR after fresh ET^b^**	Low LH^*^	0.577 (0.394, 0.843)	0.005	0.532 (0.353, 0.800)	0.002
	P level at trigger day	0.688 (0.439, 1.078)	0.103	0.601 (0.369, 0.981)	0.042
	Endometrial thickness	1.170 (1.055, 1.296)	0.003	1.191 (1.065, 1.331)	0.002
	No of embryo transferred	1.932 (1.192, 3.130)	0.008	2.014 (1.195, 3.397)	0.009
**CLBR in conventional fresh/frozen ET cycles^c^**	Low LH^*^	0.622 (0.423, 0.914)	0.016	0.596 (0.408, 0.917)	0.017
	No of oocytes retrieved	1.107 (1.056, 1.162)	<0.001	1.108 (1.055, 1.164)	<0.001
**LBR after FET^d^**	Low LH^*^	0.922 (0.722, 1.178)	0.517	0.918 (0.711, 1.183)	0.508
**CLBR in freeze-all cycles^e^**	Low LH^*^	0.896 (0.690, 1.165)	0.414	0.864 (0.652, 1.144)	0.307
	No of oocytes retrieved	1.091 (1.068, 1.115)	<0.001	1.081 (1.053, 1.110)	<0.001

^*^To evaluate the LH effect on pregnancy outcomes, the higher LH group was taken as reference. ^a^Adjusted for ET strategy (fresh vs. freeze-all), female age, infertility duration, baseline FSH and LH levels, total Gn dose, dose of GnRH antagonists and rLH supplementation, E_2_ levels at trigger day, and oocytes count. ^b^Adjusted for dose of GnRH antagonists, progesterone and LH levels at trigger day, endometrial thickness at trigger day, and number of embryos transferred. ^c^Adjusted for dose of GnRH antagonists, type and duration of infertility, baseline LH levels, E_2_ and LH levels at trigger day, and oocytes count. ^d^Adjusted for the total Gn dose, dose of rLH supplementation, E_2_ levels at trigger day, oocytes count, number and stage of embryos transferred. ^e^Adjusted for female age, duration of infertility, baseline FSH and LH levels, dose of Gn and rLH supplementation, E_2_ levels at trigger day, and oocytes count.

CLBR, cumulative live birth rage; LBR, live birth rate; FET, frozen-thawed embryo transfer.

## Discussion

The present study assessed the association between serum LH levels and the CLBR per oocyte retrieval cycle and LBR after the initial ET in reproductive-aged normogonadotrophic women in GnRH antagonist IVF/ICSI cycles, in consideration of different ET strategies, for the first time. Our results suggest that low LH levels significantly decrease the likelihood of CLBs by utilizing all fresh and frozen embryos from one stimulated cycle. Furthermore, the harmful effect of low LH mainly exists in fresh embryo transfers.

In this study, we divided the patients into low and higher LH groups arbitrarily at the cut-off value of 4 IU/L according to our former experience (21). We found that some patients with low LH levels (<4 IU/L) throughout COS have no LH surge through our observations using frequent LH measurements during COS. Moreover, the routine administration of GnRH antagonists might compromise their pregnancy outcomes.

We have already known that profoundly LH suppression is detrimental for patients undergoing either GnRH agonist or GnRH antagonist-treated cycles (17, 18), and added LH is beneficial for this specific population (8). In this study, we recorded the frequency of LH under 1.2 IU/L (23) and discovered that the proportion of patients who showed LH < 1.2 IU/L at least once was higher in the low LH group (59.8% vs. 39.2%,* P*<.001). Among them, the proportion of patients with frequencies of LH < 1.2 IU/L more than twice was also higher in the low LH group (26.1% vs. 9.0%, *P*<.001). These observations suggested that patients who presented low LH levels were more likely to suffer excessive LH suppression. We used fewer GnRH antagonists and more rLH supplementation in the low LH group than those in the higher LH group. The duration of COS, the average number of oocytes retrieved, the follicular output rate, the number of MII oocytes, and the total number of embryos available between these two groups were comparable, indicating the quality and quantity of oocytes and embryos were similar between groups after clinical intervention. Additionally, patients in the low LH group were slightly younger than those in the higher LH group. Nevertheless, the young age and potential protection strategies of LH activity for the patients with low LH levels did not counteract the detrimental effect of low LH on reproductive outcomes, particularly in fresh transfer cycles.

While the role of LH in ovarian stimulation is universally accepted, a question remains whether serum LH levels influence pregnancy outcomes. Some authors failed to find any significant difference between different LH groups regarding the implantation rates, clinical pregnancy rates, and ongoing pregnancy rates (13, 15). However, in a recent study by Benmachiche et al. (24), low serum LH levels on the day of GnRH-agonist trigger are associated with reduced rates of live birth and increased early miscarriage rates. The variety in the definition of low LH, measurement parameters of LH, and the clinical interventions may explain this inconsistent. On the other hand, the effect of LH genotype may also be one of the reasons. In a recent study conducted in 591 IVF patients, Ku et al. (25) found a significantly lower clinical pregnancy rate among carriers of Trp8Arg polymorphism of the LH beta gene after IVF with the GnRH antagonist protocol despite a similar number of retrieved oocytes. Thus, the different proportions of patients carrying polymorphism of the LH beta gene cause discrepancies between studies.

The underlying mechanism by which low LH levels seem to reduce pregnancy rates has not been fully elucidated. We are not able to perform single nucleotide polymorphism analysis in routine clinical practice yet. But we can look for clues from the existing clinical data. Expect for the quality of embryos transferred, a slow luteinization process, a delayed corpus luteum function, or a poor endometrium receptivity will also decrease the LBR after fresh ETs. Accordingly, our data suggest that low serum LH levels during COS might affect the patients’ corpus luteum function or endometrium receptivity, then cause the asynchrony between the embryo and the endometrium, potentially resulting in implantation failure and poor reproductive outcomes.

The biological activity of LH is conferred primarily through binding to the specific LH receptor (LHCG-R), which is mainly expressed on ovarian theca, mural granulosa, and luteal cells (26). Moreover, LHCG-Rs are detected in oocytes, preimplantation embryos, and the endometrium, implying LH’s direct influences on oocyte quality, embryo growth and implantation, and corpus luteum function (9, 27, 28). The results from Tesarik et al. (29) demonstrated that endometrial maturation was disturbed in women with low endogenous LH but could be rescued by mid-cycle stimulation of the LH receptor with exogenous hCG in the absence of ovarian activity. Additionally, recent research by Bildik et al. (30) confirmed that the luteal granulosa cells of stimulated IVF cycles were less viable ex vivo, expressed LH receptor and anti-apoptotic genes at lower levels, underwent apoptosis earlier, and failed to maintain the estradiol and progesterone production in comparison to natural cycles. However, whether low LH concentrations during COS jeopardize oocyte/embryo developmental competency, endometrial receptivity, and corpus luteum function remains unknown. Further investigations are needed to explore these associations.

To the best of our knowledge, this paper is the first to investigate the association between LH levels during the entire COS process and reproductive outcomes, considering both the conventional fresh/frozen ET strategy and the freeze-all strategy. The long-term follow-up of the cohort allowed us to provide information on the most clinically meaningful outcomes, i.e., CLBR, which could reflect the utilization of all the embryos obtained from the stimulation cycle. Most importantly, our analysis revealed that low LH’s detrimental effect was pronounced only in fresh ET cycles. The utilization of elective FET has increased significantly in recent years due to the introduction of the GnRH agonist trigger protocol and improvements in cryopreservation techniques. However, whether or not the freeze-all policy should be offered to the overall IVF population remains controversial (31). Two recent well-designed RCTs demonstrated that frozen embryo transfer did not result in significantly higher live birth rates than the transfer of fresh embryos among ovulatory normal responders undergoing IVF (32, 33). This study provides new insights into LH’s role on the endometrium and corpus luteum function from stimulation cycles. Meanwhile, we raise the question of whether or not serum LH concentration could be a biomarker that may help clinicians manage the process of ovarian stimulation and provide consultation in terms of embryo transfer for infertile couples.

The main weakness of this study is its retrospective nature. To minimize confounding potential, we strictly selected patients according to inclusion and exclusion criteria and adjusted our analysis for multiple variables. The confounding factors, either previously known to affect LBRs or varied significantly between the study groups, were all mentioned. Only the variables showing a tendency of association with CLBs or LBs in the univariate analyses (*P*<.25) were entered in the final multivariate model. Additionally, this study consists of a sufficient sample size (n=1480) to satisfy the multivariate analysis of these variables and correct for confounding factors. Second, the exogenous hormonal agents used for COS were not homogeneous in this study. The timing of rLH supplementation varied based on the day of GnRH antagonist administration. Though fewer GnRH antagonists and more rLH supplementation were used for patients with low LH levels, indicating an apparent insufficient LH for this subgroup of patients, the use of a homogeneous exogenous gonadotrophin might clarify the effect of LH on IVF outcome. Besides, we distinguished patients by an arbitrarily chosen LH level. LH percentiles should be calculated to achieve a more precise cut-off value. However, when we stratified patients according to the 5^th^ or 10^th^ LH peak level, the sample size was relatively small in patients with low LH levels in the conventional ET cycles. The sample imbalance between the two LH groups will significantly reduce the efficiency of statistical testing and analysis. Lastly, this study was conducted in normogonadotrophic patients with at least one available embryo formed. The conclusions cannot be extrapolated to the general population. Therefore, further prospectively designed studies with larger samples are needed to reinforce our findings.

In summary, our study demonstrated that LH levels during COS with the GnRH antagonist protocol were individualized, and the low serum LH levels were associated with a decreased CLBR per oocyte retrieval cycle and LBR after fresh ET. Currently, individually tailored ovarian stimulation is advocated. Although needing further confirmation, our results indicate that more attention should be paid to LH levels and activities, especially when the follicular development was asynchronous with serum hormone profiles when performing COS, to adjust the medication regimen accordingly and to aim towards LH levels in an optimal range in an attempt to maximize the reproductive outcomes. In the future, basic research focusing on different LH levels in assisted reproduction is needed. Meanwhile, whether the freeze-all strategy was superior to the fresh transfer strategy for patients with improper LH levels remains to be examined.

## Data Availability Statement

The raw data supporting the conclusions of this article will be made available by the authors, without undue reservation.

## Ethics Statement

The studies involving human participants were reviewed and approved by the Ethics Committee of Chao-Yang Hospital. The patients/participants provided their written informed consent to participate in this study.

## Author Contributions

YYL contributed to data collection, analysis and interpretation, manuscript drafting, and critical discussion. SL contributed to the critical discussion and revision of the manuscript. LH contributed to the analysis and interpretation of the data. HS and YHR contributed to the critical discussion of the manuscript. HML, TYW, and HHL contributed to the data collection and revision of the manuscript. YL contributed to the study design, supervision, interpretation of data, and critical discussion of the manuscript. All authors contributed to the article and approved the submitted version.

## Funding

This study was supported by the 2018 Fertility Research Program of Young and Middle-aged Physicians-China Health Promotion Foundation.

## Conflict of Interest

The authors declare that the research was conducted in the absence of any commercial or financial relationships that could be construed as a potential conflict of interest.

## Supplementary Material

Supplementary Figure 1 Distribution of the categories of the number of oocytes retrieved (<4, 4-9, 10-15, >15) of the low and higher LH groups. **(A)** For conventional fresh/frozen embryo transfer cycles. **(B)** For the freeze-all cycles.
