# Supplementary figures and images for: Low Serum LH Levels During Ovarian Stimulation With GnRH Antagonist Protocol Decrease the Live Birth Rate After Fresh Embryo Transfers but Have No Impact in Freeze-All Cycles
Source: Front Endocrinol (Lausanne). 2021 Apr 23;12:640047. doi: 10.3389/fendo.2021.640047 (PMC8104121; doi:10.3389/fendo.2021.640047)

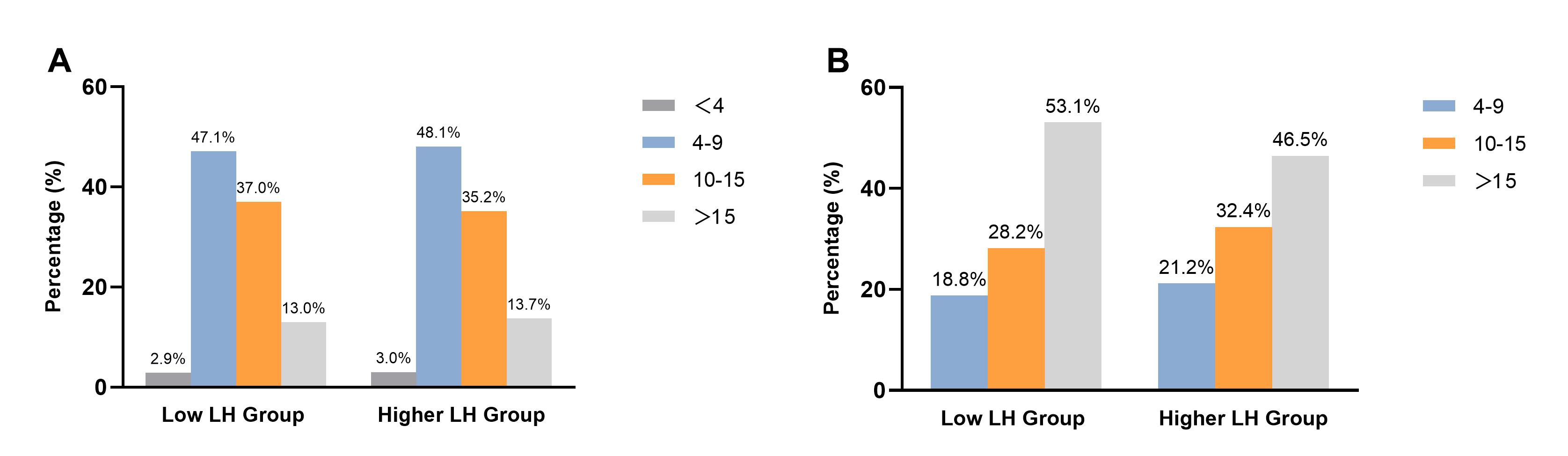

Supplement: Supplementary Figure 1 — Distribution of the categories of the number of oocytes retrieved (<4, 4-9, 10-15, >15) of the low and higher LH groups. (A) For conventional fresh/frozen embryo transfer cycles. (B) For the freeze-all cycles. [file Image_1.jpeg]
