# Supplementary material for: Low Serum LH Levels During Ovarian Stimulation With GnRH Antagonist Protocol Decrease the Live Birth Rate After Fresh Embryo Transfers but Have No Impact in Freeze-All Cycles
Source: Front Endocrinol (Lausanne). 2021 Apr 23;12:640047. doi: 10.3389/fendo.2021.640047 (PMC8104121; doi:10.3389/fendo.2021.640047)
Supplement: Supplementary file 2 [file Table_1.docx]

**Supplementary Table 1. Patient and cycle characteristics in fresh transfer and freeze-all cycles.**

|  | **Fresh cycle** | | | **Free-all cycle** | | |
| --- | --- | --- | --- | --- | --- | --- |
|  | **Group A, n=208** | **Gropu B, n=233** | ***P*** | **Group A, n=458** | **Gropu B, n=581** | ***P*** |
| **Age** | 31.55 ± 3.54 | 32.28 ± 3.87 | 0.039 | 31.45 ± 3.64 | 32.14 ± 3.73 | 0.003 |
| **BMI** | 22.92 ± 3.42 | 22.80 ± 3.68 | 0.722 | 22.76 ± 3.41 | 22.86 ± 3.60 | 0.647 |
| **AFC** | 11.99 ± 4.84 | 12.86 ± 5.65 | 0.086 | 17.53 ± 8.00 | 17.21 ± 7.96 | 0.525 |
| **Duration of infertility (y)** | 3 (2, 4) | 2.5 (2, 4) | 0.748 | 2 (2, 4) | 3 (2, 4) | 0.743 |
| **E_2_ at baseline (pg/mL)** | 47.72 ± 19.77 | 49.38 ± 19.64 | 0.378 | 48.06 ± 18.66 | 49.13 ± 17.80 | 0.347 |
| **FSH at baseline (IU/L)** | 7.26 ± 1.74 | 7.09 ± 1.92 | 0.347 | 6.35 ± 2.09 | 6.65 ± 1.88 | 0.015 |
| **LH at baseline (IU/L)** | 3.36 ± 1.91 | 4.24 ± 1.96 | <0.001 | 4.15 ± 2.92 | 4.96 ± 2.38 | <0.001 |
| **Total gonadotrophin dose (IU)** | 2389.07 ± 764.45 | 2389.06 ± 741.60 | >0.5 | 2255.97 ± 796.57 | 2303.50 ± 827.83 | 0.35 |
| **Duration of stimulation (d)** | 9.58 ± 1.49 | 9.71 ± 1.37 | 0.335 | 10.00 ± 1.44 | 9.80 ± 1.41 | 0.028 |
| **Total antagonist dose (mg)** | 0.375 (0.25, 0.5) | 0.5 (0.25, 0.75) | <0.001 | 0.375 (0.125, 0.519) | 0.5 (0.375, 0.625) | <0.001 |
| **Added rLH dose (IU)** | 487.5 (150, 975) | 300 (75, 900) | 0.016 | 375 (150, 825) | 225 (75, 675) | <0.001 |
| **E_2_ at trigger day (pg/mL)** | 2505.83 ± 1184.88 | 2564.70 ± 1301.43 | 0.621 | 4117.46 ± 2254.28 | 3991.93 ± 2425.63 | 0.393 |
| **P at trigger day (ng/mL)** | 0.75 ± 0.48 | 0.80 ± 0.40 | 0.241 | 0.99 ± 0.61 | 1.02 ± 0.77 | 0.518 |
| **LH at trigger day (IU/L)** | 2.28 ± 0.89 | 3.32 ± 2.00 | <0.001 | 2.03 ± 1.06 | 3.05 ± 1.88 | <0.001 |
| **FORT ^a^** | 0.54 ± 0.23 | 0.49 ± 0.21 | 0.04 | 0.54 ± 0.25 | 0.53 ± 0.25 | 0.41 |
| **Insemination method** | | | | | | |
| **IVF** | 142 (68.3%) | 167 (71.7%) | 0.436 | 312 (68.1%) | 370 (63.7%) | 0.135 |
| **ICSI or IVF - ICSI split** | 66 (31.7%) | 66 (28.3%) |  | 146 (31.9%) | 211 (36.3%) |  |
| **Fertilization rate (IVF)** | 0.63 ± 0.22 | 0.66 ± 0.24 | 0.239 | 0.62 ± 0.21 | 0.57 ± 0.24 | 0.004 |
| **Fertilization rate (ICSI)** | 0.71 ± 0.22 | 0.75 ± 0.23 | 0.311 | 0.75 ± 0.19 | 0.76 ± 0.20 | 0.576 |
| **No. of oocytes retrieved** | 10.24 ± 4.61 | 10.17 ± 4.43 | 0.882 | 16.38 ± 7.00 | 15.83 ± 7.42 | 0.22 |
| **No. of MII oocytes** | 7.33 ± 3.77 | 7.24 ± 3.73 | 0.889 | 10.98 ± 5.95 | 10.29 ± 5.84 | 0.278 |
| **No. of good-quality embryos on day 3** | 3.18 ± 2.68 | 3.42 ± 2.49 | 0.335 | 4.79 ± 3.70 | 4.56 ± 3.79 | 0.328 |
| **No. of total embryos available** | 3.47 ± 1.97 | 3.55 ± 1.91 | 0.654 | 4.58 ± 2.50 | 4.44 ± 2.50 | 0.358 |
| **Parameters in the first embryo transfer cycle** | | | | | | |
| **Endometrial thickness** | 10.19 ± 1.78 | 9.82 ± 1.95 | 0.037 | 9.28 ± 1.98 | 9.27 ± 1.82 | 0.91 |
| **No. of embryos transferred** | 1.93 ± 0.41 | 1.99 ± 0.41 | 0.165 | 1.96 ± 0.25 | 1.96 ± 0.28 | 0.97 |
| **Embryo stage at transfer** | | | | | | |
| **Cleavage** | 202 (97.1%) | 233 (100%) | 0.011 | 397 (86.7%) | 498 (85.7%) | 0.654 |
| **Blastocyst** | 6 (2.9%) | 0 |  | 61 (13.3%) | 83 (14.3%) |  |

***Note*:** Data are mean (SD), median (IQR), or n (%). E_2_= oestradiol, P=progesterone, MII=metaphase II, rLH= recombinant LH. ^a^ FORT= follicle output rate, calculated as the number of 16-22 mm preovulatory follicles/the number of 3-8 mm antral follicles on the third day of the menstrual cycle.
